# Fetal Valproate Syndrome with Limb Defects: An Indian Case Report

**DOI:** 10.1155/2016/3495910

**Published:** 2016-11-24

**Authors:** Manisha Goyal, Ashok Gupta, Manish Sharma, Priyanshu Mathur, Naresh Bansal

**Affiliations:** ^1^Rare Disease Clinic, Department of Pediatrics, SMS Medical College and Hospitals, Jaipur, Rajasthan, India; ^2^Accident Emergency, Department of Pediatrics, SMS Medical College and Hospitals, Jaipur, Rajasthan, India; ^3^Department of Pediatrics, SMS Medical College and Hospitals, Jaipur, Rajasthan, India

## Abstract

Epilepsy is a common disorder and exposure to antiepileptic drugs during pregnancy increases the risk of teratogenicity. Older AEDs such as valproate and phenobarbital are associated with a higher risk of major malformations in the fetus than newer AEDs like lamotrigine and levetiracetam. Exposure to valproic acid during first trimester can result in fetal valproate syndrome (FVS), comprising typical facial features, developmental delay, and a variety of malformations such as neural tube defects, cardiac and genitourinary malformations, and limb defects. We are presenting an Indian case of FVS with major limb defects.

## 1. Introduction

Valproic acid (VPA) is a commonly used drug for the management of epilepsy and certain mood disorders. It is still a common drug for epilepsy during pregnancy, despite increased awareness of its teratogenicity. The teratogenic effects of VPA were first recognized in 1980 [1]. Exposure to valproic acid during first trimester can result in fetal valproate syndrome (FVS). It comprises typical facial features (trigonocephaly, epicanthal folds, infraorbital groove, medial deficiency of eyebrows, broad nasal root, and short nose with anteverted nares, shallow philtrum, long thin upper lip, and small mouth), developmental delay, and a variety of malformations such as neural tube defects, cardiac and genitourinary malformations, and limb defects [2]. A variety of limb defects have been reported with FVS. Here, we report a case of FVS with major limb defects in an Indian boy, born of an epileptic mother who had taken sodium valproate during her pregnancy.

## 2. Case Summary

**Figure 1:**
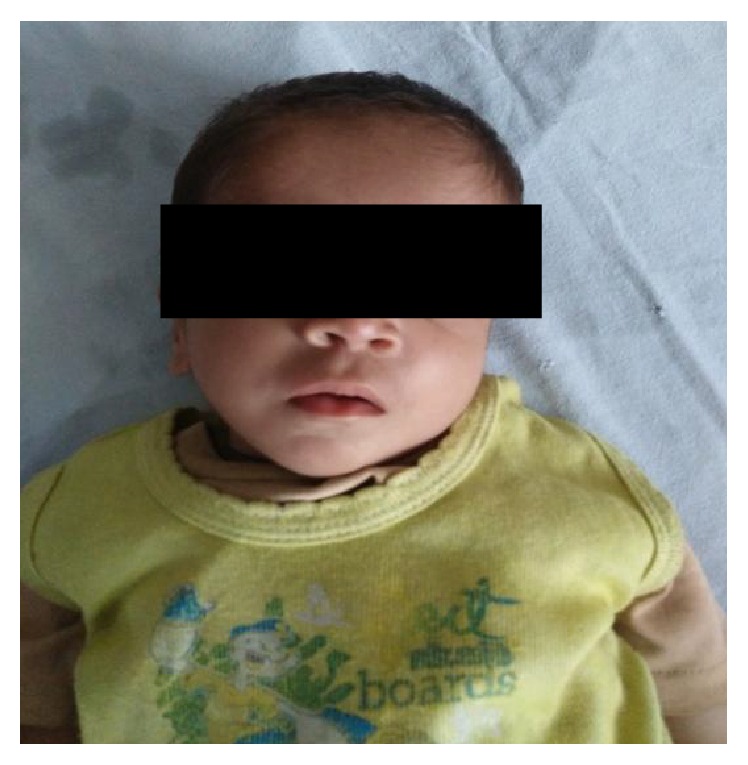
Facial features show bifrontal prominence, high forehead, left epicanthal fold, depressed and broad nasal bridge, upturned nares, prominent philtrum, thin upper lip, and bilateral low set ears.

**Figure 2:**
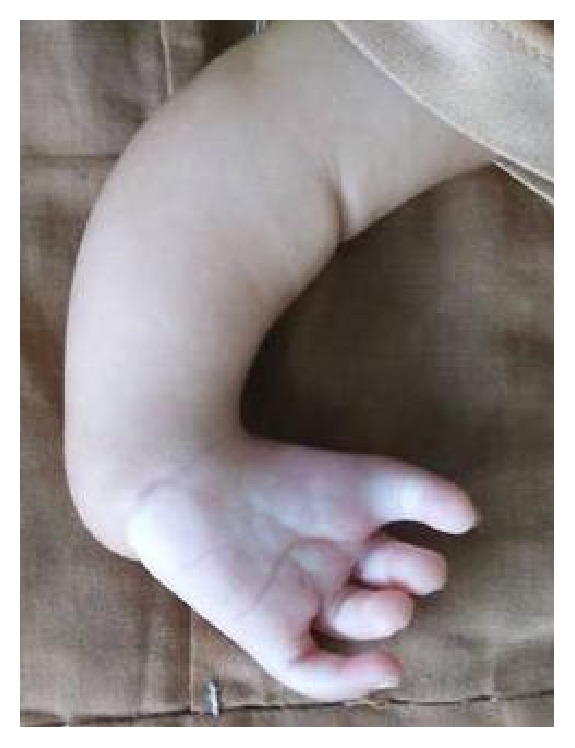
Left upper limb, short left forearm, absent thumb, ulnar deviation at wrist, and contracture of fingers at PIP joint.

**Figure 3:**
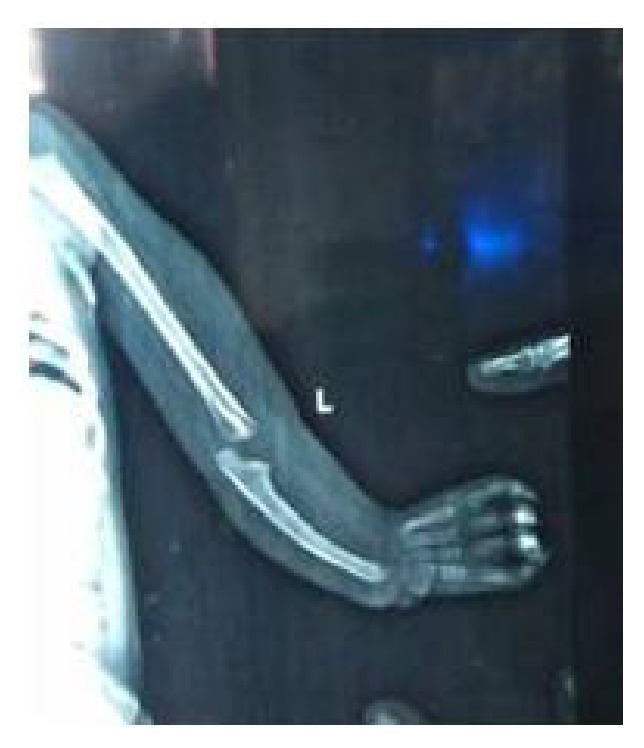
X-rays left hand: absent radius with absent thumb bone.

An eleven-month-old boy came to our genetic clinic with the complaints of difficulty in breathing. He was second in birth order of second degree consanguineous couple at term after normal vaginal delivery with birth weight of 3 kg. Baby was hospitalized for 5 days for delayed cry at birth. His mother has history of epilepsy and was on sodium valproate since last 10 years. She had taken 1000 mg of valproate in two divided doses throughout pregnancy. Baby has global developmental delay as there is no neck holding and no speech or interaction with parents at 11 months of age. His weight, length, and head circumference were 5 kg (less than 3rd centile), 67 cm (less than 3rd centile), and 40.2 cm (less than 3rd centile), respectively. Facial examination revealed bifrontal prominence, high forehead, left epicanthal fold, depressed and broad nasal bridge, upturned nares, prominent philtrum, thin upper lip, posterior cleft palate, and bilateral low set ears (Figure 1). Limb examination detected shortening of left forearm, absent thumb, ulnar deviation at wrist and contracture of 1st, 2nd, and 3rd fingers at PIP joints of left hand (Figure 2), and rudimentary thumb with contracture at proximal phalangeal joint of fingers of right hand. Overriding of toes was present on right foot. There was no hepatosplenomegaly nor were there any neurocutaneous stigmata. Genital examination showed absent testis on left side. His body tone was normal with normal reflexes. X-ray hands were detected with absent radius and with absent thumb bone on left side (Figure 3). Complete blood counts, renal function tests, liver function test, and hearing evaluation were normal. Ultrasonography abdomen showed presence of left testis in inguinal canal. 2D echocardiography was suggestive of ostium secundum atrial septal defects of 1.6 mm size. A diagnosis of fetal valproate syndrome was made on the basis of antenatal history of valproate intake, facial dysmorphism, radial ray defect, cardiac defect, and associated genital anomaly.

## 3. Discussion

Epilepsy is a common disorder and exposure to antiepileptic drugs during pregnancy increases the risk of teratogenicity. The absolute risk of major malformations in infants exposed to antiepileptic drugs is quoted as 7–10% as compared to 2-3% rate of major malformations in the general population [3]. The risk is dependent on dose and duration of drug and exposure to single drug or multidrug. Study by Tomson et al. suggested that the risk of major congenital malformations is influenced not only by type of antiepileptic drug but also by dose and other variables. They assessed rates of major congenital malformations in 1402 pregnancies exposed to carbamazepine, 1280 on lamotrigine, 1010 on valproic acid, and 217 on phenobarbital. They concluded that lamotrigine (less than 300 mg per day) and carbamazepine (less than 400 mg per day) are associated with lowest rates of malformation. They found higher risk with valproic acid and phenobarbital as compared with other antiepileptic drugs [4]. UK epilepsy and pregnancy registry has published their fifteen-year prospective observational data of intrauterine exposure to valproate, carbamazepine, and lamotrigine. They reported that in utero exposure to valproate carries a significantly higher malformation risk than lamotrigine and carbamazepine monotherapy [5]. Similar observation is reported by North American AED pregnancy registry (Hernández-Díaz et al.) as traditional AEDs such as valproate and phenobarbital are associated with a higher risk of major malformations in the fetus than newer AEDs like lamotrigine and levetiracetam [6].

Study by Morrow et al. showed that those exposed to more than 1000 mg of valproate had the highest risk of major deformities compared to other monotherapy drugs. Carbamazepine had the lowest risk of malformation as monotherapy drug. The risk is higher for polytherapy than for monotherapy. Polytherapy regimens containing valproate had a higher risk of malformation [7]. In our case mother was taking 1000 mg sodium valproic acid per day since last 10 years before conception and continued taking the same dose throughout pregnancy.

The mechanism(s) to produce teratogenicity by a specific antiepileptic drug remains undiagnosed. Some animal studies using inbred strains of mice suggested genetic background for the risk of teratogenicity [8]. Study by Dean et al. showed that the malformations found in fetal anticonvulsant syndromes are associated with folic acid deficiency and methylene-tetrahydrofolate reductase (MTHFR) polymorphisms. They concluded that the risk is three to four times higher for mothers who were MTHFR 677TT homozygotes compared with MTHFR 677CC homozygotes [9].

The “fetal valproate syndrome” includes facial dysmorphism such as epicanthal folds, flat nasal bridge, small nose with anteverted nares, thin and long upper lip with relatively shallow philtrum, and small mouth. Major congenital malformations include limb defects, neural tube defects, congenital heart defects, oral clefts, and urogenital abnormalities. Other less frequent abnormalities include inguinal and umbilical hernia, supernumerary nipple, low birth weight, behavioral issues, and development delay [10]. Schorry et al. concluded in their study that FVS is associated with early motor and speech developmental delays, low normal or borderline IQ, and adaptive behaviors [11]. Meador et al. did a prospective multicenter study of cognitive effects of fetal exposure to commonly used antiepileptic drugs (carbamazepine, lamotrigine, phenytoin, or valproate) in children aged 3 years and 4.5 years. They found that children with fetal exposure to valproate had reduced IQ at 6 years compared with other commonly used antiepileptic drugs. Valproate exposure was also associated with worse verbal and memory abilities compared with the other antiepileptic drugs and worsened nonverbal and executive functions compared with lamotrigine [12].

The risk of a limb abnormality from VPA exposure has been estimated to be about 0.42% [9]. A variety of limb defects are reported in FVS including preaxial and postaxial polydactyly, clinodactyly, talipes, broad big toes and fingerlike thumbs, contractures of the fingers, arachnodactyly, overlapping digits and syndactyly, radial ray aplasia or reduction, and phocomelia [13, 14]. In our case radial ray defect was present in the form of unilateral absence of radius and thumb along with rudimentary thumb on contralateral side. Sharony et al. showed that the upper extremities are more severely and frequently affected than the lower extremities in FVS [15]. They also concluded that the extent and severity of limb reduction defects can also vary from oligodactyly to total absence of upper extremity. Bilateral deficiency of upper extremities is reported by Guven et al. [14]. Genitourinary anomalies are seen relatively frequently with VPA exposure including hypospadias, undescended testes, renal hypoplasia, hydronephrosis, and duplication of the calyceal system [3, 16]. Our patient had unilateral testis. Other genital examinations were normal and no renal abnormalities were detected on the ultrasound scan.

Our case has radial ray defect in view of absent radius with absent thumb. Radial ray defect is a large spectrum of anomalies which ranges from partial (radial hypoplasia) to a complete (radial aplasia) deficiency of the radius with or without anomalies of thumb. Differential diagnoses of radial ray defects are Holt-Oram syndrome (cardiac-limb syndrome), Fanconi pancytopenia syndrome, Nager acrofacial dysostosis, thrombocytopenia-absent radius (TAR syndrome), VACTERL syndrome, Baller-Gerold syndrome, Rothmund-Thomson syndrome, Aase syndrome, Townes-Brocks syndrome, and Trisomy 13/18. In our patient blood counts for all three cell lines were normal, excluding Fanconi anemia, Aase syndrome, and TAR syndrome. Normal ears/hearing evaluation ruled out Holt-Oram, Townes-Brocks, and Nager syndromes. Absence of other characteristic malformations rules out VATER/VACTERL. Patient was not affording so a sample could not be sent for more elaborative genetic study such as array CGH or whole exome sequencing.

Management of FVS includes multidisciplinary team approach comprising management of the associated malformations. Physiotherapy and early speech therapy are essential for patients with developmental delay.

VPA is a widely used AED, particularly in poor resource countries like India, and its efficacy cannot be disputed. The balance between its therapeutic benefits and its teratogenic effects is mainstay in the management of pregnant women with epilepsy. VPA should not be routinely prescribed. If there is no effective alternative, then doses should be reduced to below 1 gram per day, administered in divided doses and in the slow release form. Also, women who take more than one medication for control of their epilepsy should be advised to change to monotherapy if possible. In addition, high dose folic acid (5 mg/day) should be given to all pregnant women with antiepileptic drugs, starting at least 6 weeks before conception and continuing through the first trimester [17].

We conclude that pregnant women on antiepileptic drugs are at increased risk for major congenital malformation. Most of these anomalies can be detected by detailed ultrasound examination. So we recommend avoidance of valproic acid if possible, supplementation with folic acid, and a detailed ultrasound to these women.
